# UNVEILING PSYCHOLOGICAL INTIMATE PARTNER VIOLENCE RISK FACTORS AMONG OLDER WOMEN

**DOI:** 10.1093/geroni/igae098.2931

**Published:** 2024-12-31

**Authors:** Rujeko Machinga-Asaolu, Kathryn Showalter

**Affiliations:** University of Kentucky, Lexington, Kentucky, United States; University of Kentucky, Lexington, Kentucky, United States

## Abstract

This conference poster presentation delves into the critical yet often overlooked issue of intimate partner violence (IPV) among older women. Despite its historical presence, IPV against older women aged 50 and above remains understudied, with less than 10% of IPV global data addressing this target population. Psychological IPV (IPVPA) emerges as the predominant form of IPV, significantly impacting the physical and mental well-being of older women. Through an integrated literature review, synthesizing recent IPV literature, this study aimed to illuminate the unique risk factors associated with IPVPA among older women worldwide. Utilizing the Preferred Reporting Items for Systematic Reviews and Meta-Analyses (PRISMA) framework, our research identified seven pertinent articles out of an initial pool of 355 from PubMed, CINAHL, PsycINFO, and Ageline databases. These articles met strict inclusion criteria focusing on women aged 50 and older, employing quantitative, qualitative, or mixed methods to explore IPVA. After synthesizing the included articles, we organized the descriptive risk factors into three distinct categories: vulnerabilities, stressors, and maladaptive outcomes, providing a comprehensive understanding of the multifaceted unique nature of IPVPA risk factors among older women. By meticulously evaluating existing recent research, we aim to shed light on the complexities of IPVPA and identify gaps in the literature, ultimately contributing to a more nuanced understanding of this pervasive issue. Through this presentation, we seek to raise awareness of the urgent need to address IPVPA among older women and advocate for targeted interventions and further research initiatives to better prevent IPVPA among older women.

